# Analysis of the p53/CEP-1 regulated non-coding transcriptome in *C. elegans* by an NSR-seq strategy

**DOI:** 10.1007/s13238-014-0071-y

**Published:** 2014-05-21

**Authors:** Derong Xu, Guifeng Wei, Ping Lu, Jianjun Luo, Xiaomin Chen, Geir Skogerbø, Runsheng Chen

**Affiliations:** 1 Laboratory of Non-coding RNA, Institute of Biophysics, University of Chinese Academy of Sciences, Beijing, 100101 China; 2 Graduate University of Chinese Academy of Sciences, Beijing, 100080 China; 3 Key Laboratory of Forest Protection, State Forestry Administration/Research Institute of Forest Ecology, Environment and Protection, Chinese Academy of Forestry, Beijing, 100091 China

**Keywords:** p53/CEP-1, *C*. *elegans*, ncRNA, removal rRNA, NSR-seq, high technical replicability

## Abstract

In recent years, large numbers of non-coding RNAs (ncRNAs) have been identified in *C. elegans* but their functions are still not well studied. In *C. elegans*, *CEP*-1 is the sole homolog of the p53 family of genes. In order to obtain transcription profiles of ncRNAs regulated by CEP-1 under normal and UV stressed conditions, we applied the ‘not-so-random’ hexamers priming strategy to RNA sequencing in *C. elegans*, This NSR-seq strategy efficiently depleted rRNA transcripts from the samples and showed high technical replicability. We identified more than 1,000 ncRNAs whose apparent expression was repressed by CEP-1, while around 200 were activated. Around 40% of the CEP-1 activated ncRNAs promoters contain a putative CEP-1-binding site. CEP-1 regulated ncRNAs were frequently clustered and concentrated on the X chromosome. These results indicate that numerous ncRNAs are involved in CEP-1 transcriptional network and that these are especially enriched on the X chromosome in *C. elegans*.

**Electronic supplementary material:**

The online version of this article (doi:10.1007/s13238-014-0071-y) contains supplementary material, which is available to authorized users.

## Introduction

In vertebrates, the transcription factor p53 plays critical roles in maintaining the integrity of the genome and protects against cancer by inducing cell cycle arrest, apoptosis, and DNA repair in response to genotoxic stress (Riley et al., 2008). p53 is also involved in many cellular biological activities under normal growth and development conditions including senescence (Mondal et al., 2013), metabolic processes (Berkers et al., 2013), stem cells renewal (Molchadsky et al., 2008), cell differentiation (Sabapathy et al., 1997), cell migration and invasion (Roger et al., 2006), angiogenesis (Zhang et al., 2000), autophagy regulation (Kenzelmann Broz et al., 2013; Maiuri et al., 2010), microRNA processing (Suzuki et al., 2009), immune response (Lowe et al., 2013), cell communication (Yu et al., 2006), and maternal reproduction (Levine et al., 2011). Two homologs p63 and p73 share a high degree of structural similarity and sequence identity with p53, and can also bind to the DNA promoter regions of the majority of p53 target genes (Melino et al., 2003; Yang and McKeon, 2000; Yang et al., 2010). p63 and p73 can thus directly transactivate p53-responsive genes, or function along with p53 in a variety of biological processes (Boominathan, 2010; Flores et al., 2002; Green and Chipuk, 2006; Jung et al., 2001; Levine et al., 2011; Melino et al., 2003). Although the functional repertoire of the three p53 family members shows considerable overlap, p63 and p73 have also distinct and unique biological functions. Studies have shown that p73 can regulate neural stem cell maintenance (Agostini et al., 2010), and the overexpression of transactivation-deficient p73 proteins resulted in the proliferation of human and mouse tumor cells, indicating oncogenic activity of truncated p73 isoforms (Stiewe et al., 2002). p63 is critical for maintaining epithelial development and morphogenesis (Senoo et al., 2007). These experimental evidences indicate the functional diversity of the p53 family members. The transcriptional response of target genes to p53 can be either activation or repression. It is well known that p53 transcriptionally activate genes that contain a p53 binding site, but the underlying mechanism for transcriptional repression by p53 has remained largely unexplored (Ho and Benchimol, 2003; Huarte et al., 2010; Leonova et al., 2013; Menendez et al., 2009; Riley et al., 2008).

*C. elegans* differs from mammals in that it encodes a single p53-like gene, *CEP-*1, which is considered as a pivotal transcriptional activator of the genes *EGL*-1 and *CED*-13, thereby inducing germ cell apoptosis and maintaining genome stability (Derry et al., 2001; Schumacher et al., 2001). CEP-1 contains a composite domain of an OD (oligomerization domain) and a sterile alpha motif (SAM) domain which is retained in p63 and p73 but absent in p53, and forms dimers instead of tetramers as the vertebrate p53 family members (Ou et al., 2007). Experimental evidence has also shown that CEP-1 regulates hundreds of genes during normal growth and development as well as under genotoxic stresses, and many of these genes contain a CEP-1-binding site (Derry et al., 2007). These CEP-1 regulated genes have a considerable overlap compared with their human orthologues in that they are activated or repressed by p53, p63, or p73 (Derry et al., 2007). It is thus reasonable to assume that CEP-1 is a representative of the primordial p53 family member that precedes among the vertebrate forms, and the *C. elegans* CEP-1 may thus encompass gene regulatory roles that are divided among the three vertebrate p53 family member (Derry et al., 2007; Ou et al., 2007).

In vertebrates, a rapidly increasing number of long non-coding RNAs (lncRNAs >200 bp) have been identified and functionally annotated (Guttman et al., 2009; Guttman and Rinn, 2012; Huarte and Rinn, 2010; Rinn et al., 2011), among which some are regulated by p53. The p53-activated lincRNA-p21 plays a role as a transcriptional repressor in the p53 gene regulatory network (Huarte et al., 2010). Another lncRNA, PANDA, which is located ~5kb upstream of the *CDKN1A* (p21) transcription start site, is induced by DNA damage in a p53-dependent manner and interacts with its partner NF-YA to mediate an anti-apoptotic effect (Hung et al., 2011). The lncRNA H19, whose transcription is repressed by p53, is upregulated in many tumor types, and ectopic expression of H19 increased cell proliferation of gastric cancer cells (Adriaenssens et al., 1998; Dugimont et al., 1998; Matouk et al., 2010; Yang et al., 2012). The tumor suppressor lncRNA MEG3 stabilized the p53 protein, thereby stimulating transcription from a p53-dependent promoter and thus regulating the expression of p53 target genes (Lu et al., 2013; Zhou et al., 2007).

In *C. elegans,* a large number of non-coding RNAs have been identified. Previous work in our lab using tiling arrays has indicated the existence of several thousand small transcripts of unknown function (TUFs), many of which were expressed in a developmentally-specific manner (He et al., 2007; Wang et al., 2011). Employing the 454 GS-FLX sequencing system, we have further identified 473 novel transcripts with an intermediate (70–500 nt) size-range (is-ncRNAs) (Xiao et al., 2012). Additionally, analysis of RNA-seq data from *C. elegans* identified around 170 long intergenic ncRNAs (lincRNAs) (Nam and Bartel, 2012), and integrated analysis of microarray and sequencing data with information on conservation and secondary structure suggested the existence of more than 7000 novel ncRNAs in the *C. elegans* transcriptome (Lu et al., 2011). However, the biological functions of the majority of these newly identified ncRNAs and their possible involvement in the *CEP-*1 gene regulatory network is still largely unknown.

As one kind of genotoxic stress, UV irradiation induces DNA damage which is able to initiate multiple signaling pathways involved in cell cycle arrest, DNA repair, and apoptosis. UV-induced apoptosis has further been shown to be dependent on CEP-1 (Stergiou et al., 2007). The *C. elegans* locus ZK355.8 was originally (WS190) predicted as a protein coding gene with unknown function, but has later been reclassified as an ncRNA by coding potential assessment and conservation analysis (Li et al., 2012). The expression of ncRNA ZK355.8 increased more than 10 folds in response to UV irradiation in wild-type worms, however, this up-regulation disappeared when the gene *XPA*-1 was mutationally inactivated (Li et al., 2012). XPA-1 is a component of the nucleotide excision repair (NER) pathway that acts upstream of the CEP-1 in UV-induced apoptosis (Stergiou et al., 2007), implying that the transcriptional activation of ZK355.8 upon UV irradiation was dependent on the functional *XPA*-1 gene. UV survival assays have also shown that RNAi knockdown of ZK355.8 increased UV sensitivity of the worm, indicating that ZK355.8 is an ncRNA that is involved in the UV-induced DNA damage response pathway (Li et al., 2012).

Here, in order to obtain expression profiles of ncRNAs regulated by CEP-1, we applied RNA-seq in combination the ‘not-so-random’ hexamer priming strategy (henceforth called NSR-seq) (Armour et al., 2009) in wild-type N2 and *cep-1* mutant worms. As rRNA transcripts comprise 85%–90% of the total RNA in cells, low copy number RNA species would be very difficult to detect if rRNAs were not filtered out during sample preparation. The NSR hexamers priming strategy implies filtering out hexamers with perfect match to rRNA in an organism, thereby obtaining a primer set which ideally only amplifies non-rRNA transcripts (Armour et al., 2009). This approach will result in a relative enrichment of both polyadenylated and nonpolyadenylated transcripts (other than rRNAs), and is expected to reduce the signal-to-noise ratio in the sequence data. The results obtained indicated that a substantial number of ncRNAs in *C. elegans* are influenced by CEP-1 under normal growths as well as in response to UV irradiation. In addition, the data allowed for an estimate of the performance of the NSR sequencing strategy in *C. elegans.*

## Results

### Excellent performance of NSR-seq in *C. elegans*

**Figure 1:**
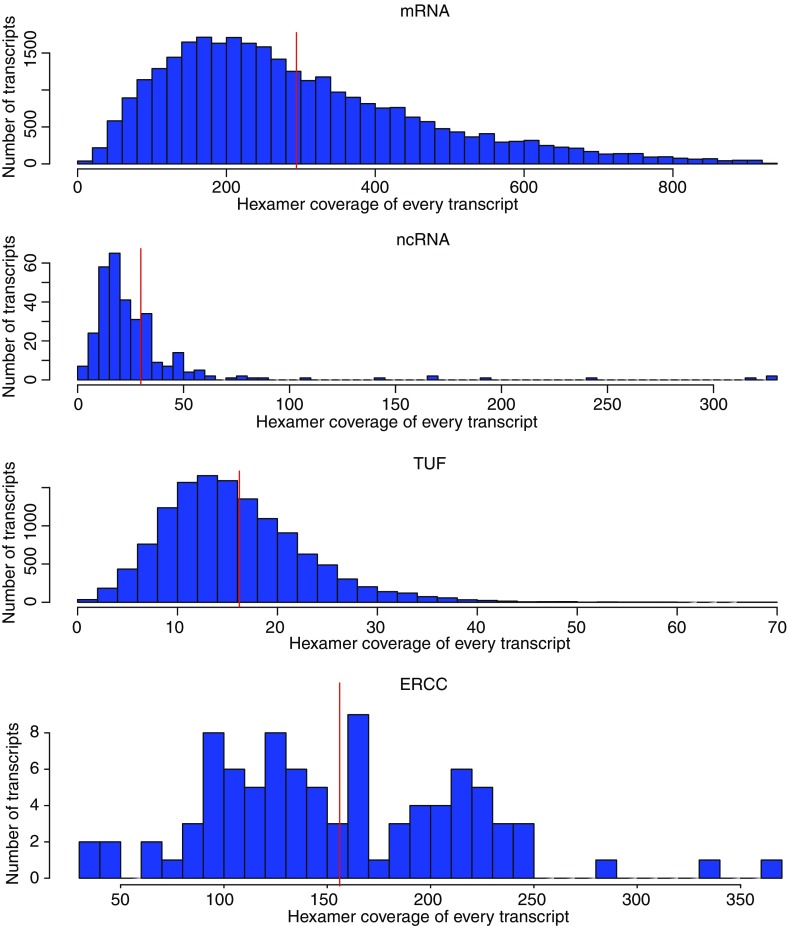
**Frequency of NSR hexamer perfectly matches mRNAs, ncRNAs, TUFs, and ERCC RNA Spike-In transcripts**. The number of perfectly matched hexamers (blue) to wormbase annotated mRNA genes (30,296), wormbase annotated intermediate-size ncRNAs (315) and our intermediate-size TUFs (12,928) and ERCC RNA Spike-In (92). The mean hexamer coverage is indicated by a red line

To date, the strand-specific NSR-seq strategy has not been used in *C. elegans*. The ‘not-so-random’ (NSR) primer set was designed by aligning a full set of random hexamer sequences to all *C. elegans* rRNA transcripts, including the cytoplasmic 18S, 28S, 5.8S, 5S rRNAs, and the mitochondrial rRNA transcripts (Table S1) (Armour et al., 2009). From the 4,096 input hexamers, 3,157 hexamers were filtered out, yielding the NSR set of 939 hexamers. This set contains 190 hexamers more than the set used for human analyses (Armour et al., 2009). In order to examine the coverage of known transcripts, the 939 NSR hexamers were next aligned to all mRNAs and ncRNAs annotated in Wormbase as well as to TUFs detected in our previous studies (He et al., 2007; Wang et al., 2011). An average of 294, 29, and 16 hexamers perfectly matched to annotated mRNAs (30,296), annotated intermediate-size ncRNAs (315) and TUFs (12,928) respectively (Fig. 1). Taking into consideration the average length of mRNAs (~2 kb), intermediate-size ncRNAs (~150 bp), and TUFs (~90 bp), this amounts to having a matching primer start site for every 5 to 6 bases of the transcripts, which indicate that the NSR primers set possess a sufficient sequence complexity to obtain high-density coverage for potential target transcripts.

Heterologous sequences were added to the 5′ ends of the hexamers to obtain the NSR primer sets (Table S2), thus allowing directional cDNA synthesis, PCR amplification and sequencing during oligonucleotide synthesis process.

**Figure 2:**
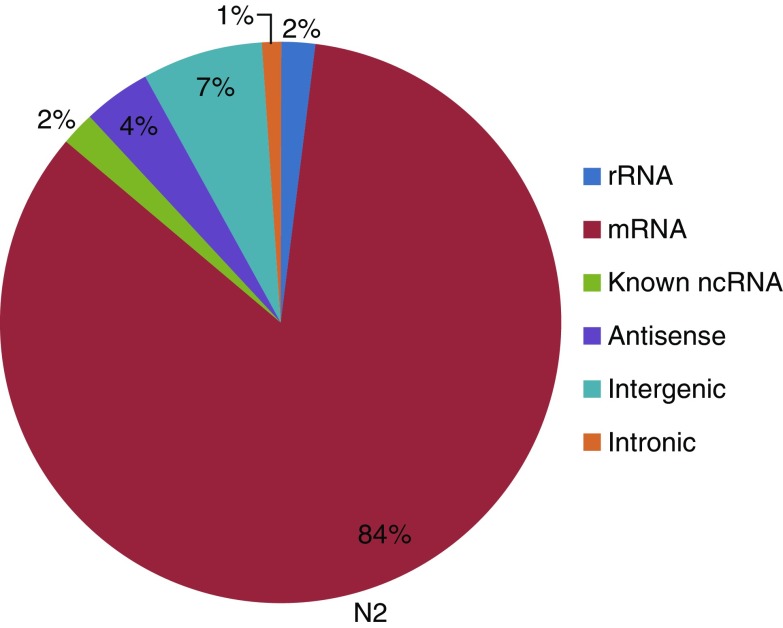
**Genomic distribution of mapped reads**. The figure shows the percentage of reads from N2 worms mapped to various genomic regions

RNA samples were extracted from wild-type (N2) and *cep-1* deficient mutant (gk138) worms which were either untreated or treated with UV irradiation at a dosage of 120 J/m^2^. The cDNA synthesis employing the NSR primer set was performed on all four RNA samples before sequencing on the IIllumina GA2 platform, producing single-end 80-nt reads. Approximately 30 million reads were generated from each sequencing sample, of which 58.10%–65.42% could be mapped to the *C. elegans* genome (WS190) with two or less mismatches (Table S4). In total, around 80% (81.39%–83.82%) of the reads were mapped to known mRNAs, and about 2.3% (2.20%–2.38%) were mapped to known ncRNAs. The remaining reads (approximately 12%) were mapped to antisense strand of coding exons (4.13%–6.18%), to introns (0.88%–1.60%) or to unannotated intergenic regions (6.38%–6.78%) (Fig. 2 and Table 1).

**Table 1:** Genomic distribution of mapped reads in all samples.

Sample	rRNA	mRNA	Known ncRNA	Antisense	Intergenic	Intronic
N2	1.97%	83.82%	2.30%	4.13%	6.78%	0.99%
N2/UV	3.30%	82.13%	2.32%	4.66%	6.72%	0.88%
cep-1	2.97%	82.16%	2.20%	4.98%	6.74%	0.95%
cep-1/UV	2.76%	81.39%	2.29%	6.18%	6.50%	0.88%
cep-1_rep	2.34%	81.59%	2.38%	5.71%	6.38%	1.60%

cep-1_rep is a biological replicate cep-1

Only about 3% (1.97%–3.30%) of all reads were mapped to rRNA transcripts (Fig. 2 and Table 1), whereas 13% of reads were mapped to rRNA loci shown in a study previously carried out in human (Armour et al., 2009), indicating that the NSR-seq strategy performs more efficiently in *C. elegans* than in human on the depletion of rRNA transcripts.

### Estimate of the NSR priming bias

**Figure 3:**
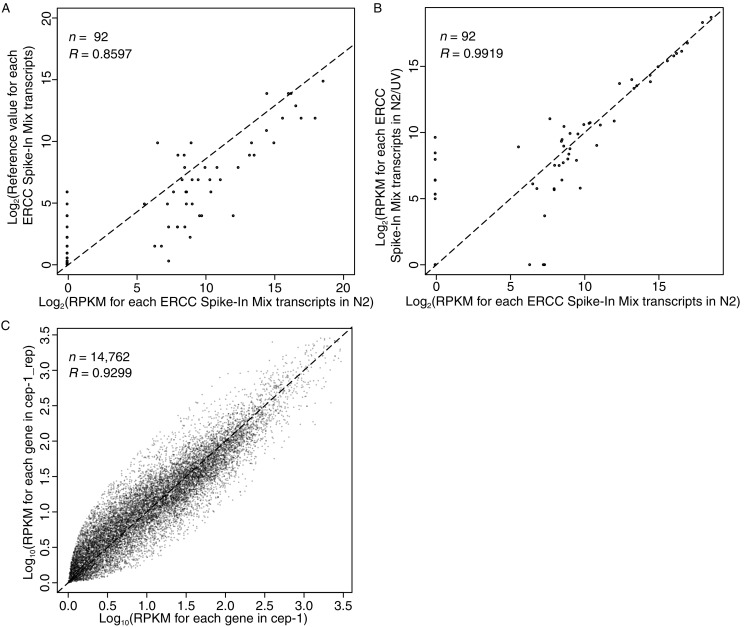
**Correlation between Spike-In Mix reference values and observed RPKM values in sequencing samples**. (A) Correlation between Spike-In Mix reference values and observed RPKM values in N2 worms under normal conditions. (B) Correlation between Spike-In Mix RPKM values from N2 worms with and without UV treatment. (C) Correlations between RPKM values of all genes from two biological replicates of *cep-1* mutant (gk138) worms

The PCR amplification step is a major source of bias during library preparation (Aird et al., 2011; Roberts et al., 2011). To evaluate the NSR-seq performance in *C. elegans*, we used the ERCC Mixes (Jiang et al., 2011; Loven et al., 2012), which are pre-formulated sets of 92 polyadenylated transcripts with moderate GC content spanning 250–2000 nt in length and a 106-fold concentration range. Incorporation of the Spike-In RNA mixes provides a set of external RNA controls that enable performance assessment on a variety of technology platforms, including next-generation sequencing (NGS), microarrays, and PCR-based assays. There were 155 hexamers in the NSR primers set that perfectly matched to at least one of the 92 Spike-In transcripts (Fig. 1), and about 0.5% of all reads mapped to Spike-In transcripts (Table S5). In order to estimate the bias introduced by the NSR primer set, all mapped reads were normalized to reads per kilobase per million mapped reads (RPKM). The RPKM of the 92 Spike-In transcripts was then used to obtain an overall estimate of the bias generated in the NSR priming in the directional cDNA synthesis, PCR amplification and sequencing. The RPKM values of the RNA Spike-In mixes were compared to reference values for the Spike-In Mix transcripts, yielding high correlation values (Pearson’s correlation coefficient *R* = 0.86–0.89, *P* < 0.01) for all samples (Fig. 3A and Table 2). Even higher correlation values (Pearson’s *R* > 0.99, *P* < 0.01) for the Spike-In Mix transcripts were found in pairwise comparisons of the samples (Fig. 3B and Table 3), suggesting high technical reproducibility and insignificant bias generated by the NSR priming.

**Table 2:** The correlation coefficient between Spike-In Mix reference values and observed expression in all samples.

Sample	Correlation with reference values
N2	0.860**
N2/UV	0.861**
cep-1	0.890**
cep-1/UV	0.877**
cep-1_rep	0.882**

**: *P* < 0.01

**Table 3:** The correlation coefficient for Spike-In Mix transcript expression in pairwise comparisons between samples.

Sample	N2	N2/UV	cep-1	cep-1/UV
N2/UV	0.996**			
cep-1	0.991**	0.992**		
cep-1/UV	0.996**	0.997**	0.998**	
cep-1_rep	0.980**	0.970**	0.984**	0.964**

**: *P* < 0.01

We also set up a biological repeat by preparing an additional of *cep-1* mutant (gk138) worm and sequencing the extracted RNA in a different flow-cell. Both samples showed equally high Pearson’s correlation coefficients (*R* = 0.88, *P* < 0.01) to the Spike-In Mix reference values (Table 2), as well as a high correlation values (Pearson’s *R* = ~0.97, *P* < 0.01) between each other (Table 3). When all mapped reads are taken into account, the expressional correlation value (Pearson’s) of the two biological replicates reached 0.93 (*P* < 0.01) (Fig. 3C).

### A large number of ncRNAs are repressed by CEP-1 in the absence of UV irradiation

**Figure 4:**
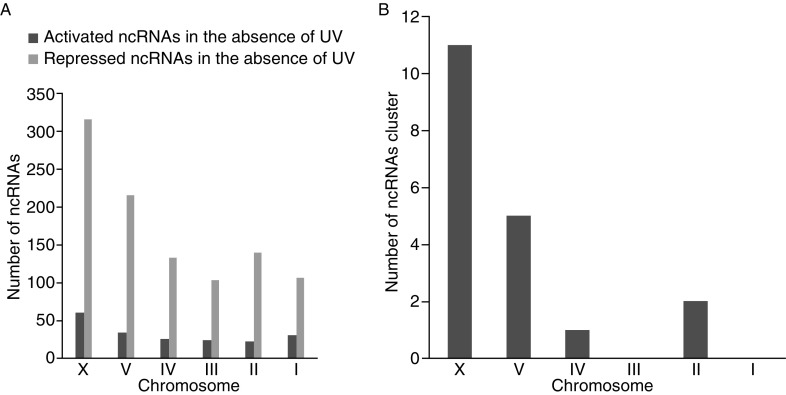
**Chromosomal distribution of CEP-1 regulated ncRNAs in the absence of UV**. (A) Chromosomal distribution of ncRNAs activated (blue) and repressed (red) by CEP-1. (B) Chromosomal distribution of 19 CEP-1 repressed ncRNA clusters

To determine whether CEP-1 regulates the activity of non-coding RNAs (>70 nt, excluding miRNAs, siRNAs, tRNAs, and etc.), we compared the expression profiles of ncRNAs from wild-type (N2) and *cep-1* mutant (gk138*)* worms under normal condition of growth and development, and identified 1209 ncRNAs (RPKM > 1) whose expression differed at least two-fold between wild-type and *cep-1* mutant. The majority of the differentially expressed ncRNAs (1014) showed higher expression in the *cep-1* mutant (i.e., in the absence of CEP-1) than in the wild-type, and only 16.1% of the ncRNAs (195) showed reduced expression in the *cep-1* mutant (Fig. 4A, Tables S6 and S7), suggesting that CEP-1 contributes more to repression than to activation of ncRNA loci.

Analysis of the genomic distribution of the differentially expressed ncRNAs showed a significant enrichment of these loci on chromosomes X and V. More than 30% of these ncRNA loci were located on chromosome X, and about 20% on chromosome V, while the remaining ncRNAs were distributed relatively evenly on chromosomes I, II, III, and IV (Fig. 4A). We also detected 19 clusters among the differentially expressed ncRNAs of which 11 and 5 were located on chromosomes X and V, respectively (Fig. 4B and Table S8). Validation of two randomly selected clusters by qRT-PCR confirmed the differential expression between wild-type and *cep-1* mutant worms under normal growth conditions (Fig. S4).

### Exposure to UV-irradiation substantially alters the ncRNA expression profiles

**Figure 5:**
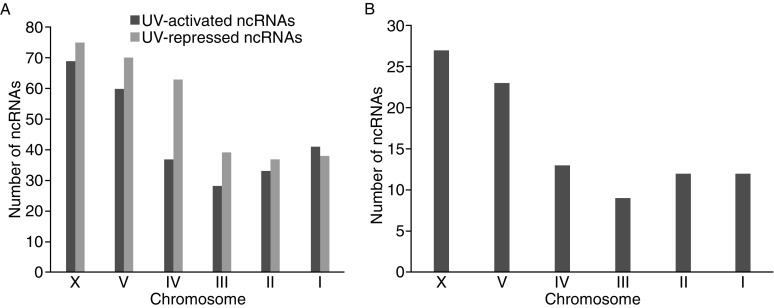
**Chromosomal distribution of ncRNAs regulated by CEP-1 in response to UV irradiation**. (A) Chromosomal distribution of ncRNA activated (blue) and repressed (red) by CEP-1 in response to UV stress. (B) Chromosomal distribution of 96 ncRNAs whose expressional status shifted from repressed to be activated in response to UV irradiation

To identify ncRNAs regulated by CEP-1 plus in response to UV stress, we treated wild-type N2 and *cep-1* mutant (gk138) worms with UV irradiation and compared their ncRNA expression profiles. In total, there were 590 ncRNAs with at least two-fold difference in expression (RPKM > 1) between wild-type N2 and *cep-1* mutant worms upon UV irradiation. Of these, 268 ncRNAs showed reduced and 322 ncRNAs showed elevated expression in the *cep-1* mutant worms when compared to the wild-type N2. Thus, the number of ncRNAs whose active expression was dependent on the presence of CEP-1 (i.e., in the wild type) almost doubled after UV irradiation (i.e., increased from 195 to 268), whereas the number of ncRNAs that were repressed by CEP-1 were reduced to about 1/3 (from 1014 to 322) after exposure to UV (Fig. 5A, Tables S9 and S10). Moreover, we also found that many of these expression-elevated ncRNAs (96 out of 268) were repressed by CEP-1 under normal growth conditions (Table S11).

**Figure 6:**
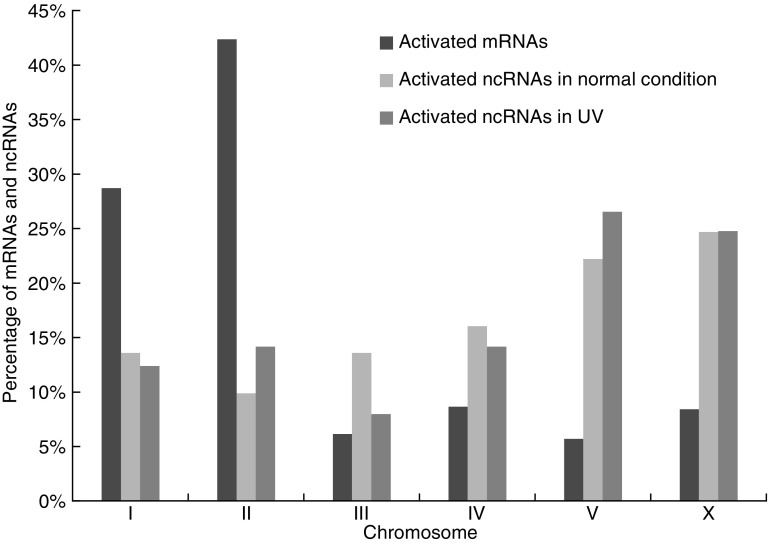
**Comparison of chromosomal distribution of mRNAs and ncRNAs activated by UV irradiation**. The activated mRNA data are from Derry et al. (2007)

In order to identify ncRNAs that may be direct targets of CEP-1, we examined the promoter regions (2-kb upstream and 500-bp downstream of the transcription start sites (TSSs)) of CEP-1 activated ncRNAs for enrichment of putative CEP-1 binding motifs RRRCWWGYYY (Huyen et al., 2004). We found that about 40% of CEP-1 activated ncRNAs contain putative CEP-1 binding motifs under normal growth conditions (81 out of 195) and in response to UV irradiation (113 out of 268) respectively (Tables S6 and S9). The activated ncRNA loci containing a putative CEP-1 binding motif were also unevenly distributed on the chromosomes in that approximately 50% of these ncRNAs were enriched on chromosomes V and X. This distribution pattern is opposite to the one detected for CEP-1 activated mRNAs (Derry et al., 2007) (Fig. 6).

### CEP-1 regulates numerous and functionally diverse ncRNAs

A number of ncRNAs of various functional categories were found to be differentially expressed in wild-type N2 and *cep-1* mutant (gk138) worms. For the purpose of validation, the differential expression of some ncRNAs was further analyzed by qRT-PCR.

**Figure 7:**
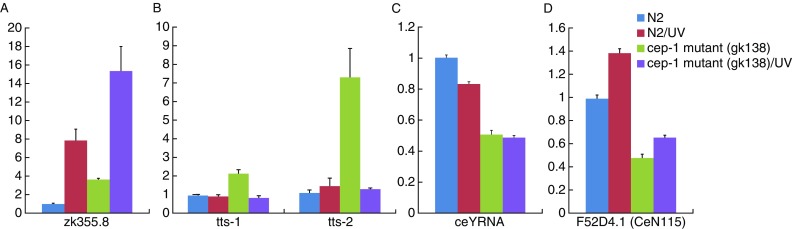
**qRT-PCR validation of differential expression of CEP-1 regulated ncRNA candidates**. The expression levels of CEP-1 regulated ncRNA candidates were examined by qRT-PCR in the wild-type N2 and *CEP-1* deletion mutant (gk138) before and after UV irradiation (120 J/m^2^). Results were normalized to the expression level of *TBG-1* and compared with the level of the wild-type without UV irradiation. Data presented are means ± SEM of at least three independent experiments. (A) ZK355.8. (B) Telomerase RNAs *TTS-1* and *TTS-2*. (C) ceY RNA. (D) F52D4.1

The ncRNA locus ZK355.8 has previously been implicated in the UV-induced DNA damage response pathway (Li et al., 2012). Our result showed that not only is the expression of ZK355.8 up-regulated more than 8 fold after UV irradiation in wild-type worms (N2/UV), but also that the expression of ZK355.8 in *cep-1* mutant (gk138) worms was about 4-fold higher than in wild-type worms, and increased 2-fold in *cep-1* mutant (gk138) relative to wild-type worms N2 after UV irradiation (Fig. 7A). Examination of the ZK355.8 promoter region identified a CEP-1 binding motif sequence (AAACATGCTC) located in 1870 bp upstream of the transcription start site (TSS), suggesting that ZK355.8 might be directly regulated by CEP-1. These results suggest that ZK355.8 is transcriptionally repressed by CEP-1 under normal growth conditions and the repressed state was abolished in response to UV irradiation.

In *C. elegans*, two putative telomerase RNAs, *TTS-1* and *TTS-2*, have been reported (Jones et al., 2001). Our data showed that in *cep-1* mutant (gk138) worms *TTS-1* and *TTS-2* were up-regulated 2-fold and 7-fold higher respectively in wild-type worms under normal growth conditions (Fig. 7B). Conserved CEP-1 binding motifs were located in 2806 bp upstream of the *TTS-1* TSS (AGGCTTGTTT) and 667 bp downstream of the *TTS-2* TSS (AAACATGTTC), respectively.

Five trans-spliced leader RNA SL2 RNAs, but not the SL1 RNA, were regulated by CEP-1 under normal growth condition or UV irradiation (Fig. S3). The expression level of ceY RNA in wild-type worms was twice that in the *cep-1* mutant, and UV irradiation did not affect the level of ceY RNA (Fig. 7C). Two out of five putative RNA components of the signal recognition particle (SRP RNAs), R144.15 and ZC155.8, and an RNase MRP RNA, *MRPR-1,* were found to be regulated by CEP-1 (Fig. S1).

SmY RNAs (or snRNA-like RNAs; snlRNAs) are only found in some nematodes, and are thought to be involved in mRNA trans-splicing by associating with the Sm protein, a component of the spliceosomal snRNPs (Jones et al., 2009; MacMorris et al., 2007; Maroney et al., 1996). F52D4.1 (CeN115) has been classified as an snlRNA (SmY RNA) (Deng et al., 2006). The expression level of F52D4.1 was two-fold higher in wild-type N2 compared to *cep-1* mutant (gk138), however, its expression was not sensitive to UV irradiation (Fig. 7D)

SnoRNAs guide the snoRNP complex to ribosomal RNA modification sites by complementary base pairing between snoRNAs and the target rRNAs (Bachellerie et al., 2002). NOLC1 is a protein component of the snoRNP, and its expression was attenuated by p53 under normal growth conditions (Krastev et al., 2011). It has thus been classified as a physiological p53 target gene, and may indicate the involvement of p53 in the snoRNP assembly pathway (Krastev et al., 2011). We also validated the differential expression of one component of the snoRNP complex, the *GAR-1* gene, which is upregulated in *cep-1* mutant (gk138) worms under normal growth conditions (Fig. S2) and contains the conserved CEP-1 binding motif in its promoter region (AAACTTGCCC, located in 2796 bp upstream of TSS). In total, 80 snoRNAs showed more than two-fold differential expression in the *cep-1* mutant (gk138) compared to wild-type N2 worms. Of these 48 were differentially expressed under normal growth conditions (6 downregulated and 42 upregulated), whereas the rest showed differential expression only in response to UV irradiation. In wild-type worms, 4 out of 80 snoRNAs shifted from repressed expression under normal growth conditions to elevated expression after UV irradiation.

## Discussion

The NSR-seq strategy has in theory many advantages and has been successfully applied in human. This approach provides a simplified procedure for the generation of high-complexity cDNA libraries based on only two steps of sequence-specific priming using the NSR primers, thus removing the need for specific steps for rRNA removal, polyadenylation selection, shearing of the input DNA, adaptor ligation, and size fractionation. In addition, this strategy enables parallel detection of polyadenylated and non-polyadenylated transcripts. Taken together, the method provides a comprehensive approach for identification and characterization of new non-polyadenylated RNA transcripts (Armour et al., 2009).

We applied the NSR-seq strategy and assessed its performance in *C. elegans*, and profiled ncRNA transcriptomes from wild-type N2 and *cep-1* mutant (gk138) worms under both normal and genotoxic stress conditions. An NSR primer set corresponding to 939 hexamers was generated, which is 190 hexamers more than the set generated for studies in human. Moreover, we included all *C. elegans* rRNA transcripts as filter sequences, while the 5.8S (156-nt) and 5S (121-nt) rRNA transcripts were excluded in human. Given the 30 folds differences in genome size between *C. elegans* (~100 Mb) and human (~3,000 Mb) (Consortium, 1998; Venter, 2001), an NSR hexamer set generated for *C. elegans* had higher coverage and better uniformity read coverage than those applied in human.

We first used Spike-In Mix transcripts to evaluate the bias generated from the NSR-seq strategy, indeed, our data show that slight bias exists in the processes of NSR priming even though NSR primer set can produce high-density coverage for potential target transcripts. The underlying explanation for this is that the NSR primer set possesses less coverage uniformity compared to the random hexamers, and the immoderate GC content distribution in the genome and in the NSR hexamers set. Inappropriate GC content in the NSR hexamers set and a less uniform NSR read coverage were found in the human studies as well, and the coverage uniformity cannot be improved by the fragmentation of RNA or replacement of NSR sequences with random hexamers before the cDNA synthesis (Armour et al., 2009). GC content and evenness GC distribution in the chromosomes are important factors influencing the bias generated by PCR based processes. The GC content (36%) of the *C. elegans* genome is slightly less than that of the human genome (GC%: 41%), and unlike the mosaic distribution of GC content in human genome, GC is essentially evenly distributed across all the chromosomes in *C. elegans* (Consortium, 1998). These evidences suggest that the NSR-seq method is more suitable in *C. elegans* than in human owing to more hexamers in the NSR set, small genome size, and more evenly GC content.

As a whole, the NSR-seq method has shown good performance in *C. elegans*, especially with respect to removal of reads corresponding to rRNAs and technical replicability. Despite of a slight bias generated during NSR hexamer priming, the NSR-seq method still robustly reproduces the same transcript sites in different samples.

Telomerase is a ribonucleoprotein complex which is required for maintaining telomere length in vertebrates, and consists of the telomerase reverse transcriptase (TERT) and the telomerase RNA (TR) (Kirkpatrick and Mokbel, 2001). Studies have showed that activation of the telomerase and inactivation of p53 is frequently detected in human cancers, and the overexpression of wild-type p53 can transcriptionally reduce the expression level of telomerase in various cancer cell lines (Xu et al., 2000). Direct interaction between p53 and telomerase was also shown in human breast cancer cells by affinity chromatography and immunoprecipitation assays *in vitro* (Li et al., 1999). These findings imply that CEP-1 might bind and transcriptionally repress the expression of telomerase RNAs and thereby reduce the telomerase activity in order to maintain chromatin stability in *C. elegans*. Interestingly, in contrast to the human telomerase RNA, which activates ATR, endogenous hTR levels increase independently of the telomerase status in response to low UV radiation (Kedde et al., 2006), UV radiation did not significantly alter the expression of *TTS-1* and *TTS-2* in *C. elegans.*

Approximately 70% of the *C. elegans* coding genes are trans-spliced by the addition of 22-nt trans-spliced leader RNA sequences at the mRNA 5′ end. The spliced leader RNAs occur in two forms in *C. elegans*, SL1 RNA and SL2 RNA (Allen et al., 2011). The majority of trans-spliced genes are donated by independent one of SL1 or SL2, indicating that SL1 and SL2 trans-splicing use different underlying mechanisms (Allen et al., 2011). The observation that five SL2 RNAs but no SL1 RNA was regulated by CEP-1 suggested that the CEP-1 has a role in trans-splicing of SL2 but not of SL1 RNA.

Y RNAs are RNA components of the Ro60 ribonucleoprotein particle, which is necessary for DNA replication through interactions with chromatin. Y RNAs required for cell proliferation are frequently overexpressed in many human tumors (Christov et al., 2008). There are four different human Y RNAs (hY1, hY3, hY4, and hY5 RNA) with different expression patterns. Only one Y RNA (YRN-1, named ceY RNA) has been found in *C. elegans*, and is apparently most closely related to the hY3 RNA (Labbe et al., 2000; Labbe et al., 1999). These results, in combination with the roles of human Y RNAs on DNA replication and cell proliferation, are compatible with the observation that ceY RNA was indirectly activated by CEP-1 to maintain DNA replication and cell proliferation under normal growth and development conditions.

CEP-1 also affects the expression of snoRNAs and the snoRNP component GAR-1, SRP RNAs, and the RNase MRP RNA *MRPR-1*, suggesting that CEP-1 may be involved in ribosome assembly, cellular proliferation, protein translocation, and SL2 trans-splicing with some underlying mechanisms.

The potentially CEP-1 regulated ncRNAs were not randomly distributed in the genome loci, but were frequently clustered and significantly enriched on the X chromosome. This bear some resemblance with CEP-1 regulated mRNA genes, which are also clustered (Derry et al., 2007). However, in contrast to the CEP-1 regulated ncRNAs, the CEP-1 regulated mRNAs are not concentrated on chromosome X (Derry et al., 2007). Reports have also shown that many p53-induced lincRNAs are able to serve as regulatory hubs to mediate global gene repression in human (Huarte et al., 2010). Together, these results suggest that similar to the findings from human p53, CEP-1 induced ncRNAs might play their roles by repressing mRNAs in the regulatory pathways.

The repressed chromatin state of the *C. elegans* X chromosomes is interspersed in *cis* over short or long distances that is maintained under normal growth and development conditions, thus regulating the global expression of the X chromosomes (Fong et al., 2002; Kelly et al., 2002). While the transcriptional activation of target genes is mediated by the direct binding of p53 to consensus sequences in their promoters, several mechanisms have been proposed for p53-mediated repression. These include sequestration of components of the basal transcriptional machinery, interfering with the functions of DNA-binding transcriptional activators, or regulation of chromatin structure at the promoters of target genes by recruiting histone deacetylases (Ho and Benchimol, 2003). Many RNA polymerase III transcribed ncRNAs are repressed by p53 with targeting TBP and inhibiting promoter occupancy by TFIIIB (Crighton et al., 2003).

In conclusion, transcriptional repression of a large number of ncRNAs by p53/CEP-1 is important for its ability to maintain chromosome stability with underlying mechanism and unknown functional consequences of transcriptional repression. On the other hand, many of these CEP-1 regulated ncRNAs were frequently enriched on the X chromosome and some of these ncRNAs were especially clustered on X chromosome.

## Materials and methods

### RNA extraction

Two *C. elegans* strains, N2 (wild-type) and the *cep-1* mutant (gk138), were used. The worms were obtained by growing synchronized L1s (L1 starved) worms on NGM plates seeded with OP50 at 20°C to young adult stage.

Total RNA was isolated from synchronized populations of N2 and *cep-1* (gk138) young adult worms using the Trizol reagent (Invitrogen) according to the manufacturer’s instructions.

### UV irradiation

Young adult larvae were treated with UV irradiation (UVC 254 nm; UVP CX-2000) at a dosage of 120 J/m^2^. RNA was extracted 4 h after treatment with radiation. Contaminant DNA was removed with DNase I (Fermentas).

### NSR primer set

According to methods described previously (Armour et al., 2009), we designed compatible amplification primer sequences for sequencing on the Illumina GAII sequencing platform. NSR hexamers were synthesized with a 5′-amplification annealing site for the first-strand (5′-TCCGATCTCTN-(NSR reverse complement)-3′) and the second strand (5′-TCCGATCTGAN-(NSR)-3′) primers. The same forward and reverse primers (Table S3) were used for PCR amplification of the NSR-primed cDNA libraries. Primers corresponding to each of the 939 hexamers in the NSR collections were synthesized individually, desalted and dissolved in nuclear-free water to 100 µmol/L. The primers were then mixed at equal-molar concentration to yield the 939 NSR primer set.

### Library generation

2 µL of a 1:100 dilution of Spike-In Mix (Ambion) were added to 1 µg of total RNA from each sample, following the manufacturer’s guidelines. NSR-primed cDNA synthesis, second-strand synthesis, and PCR amplification followed a previously described method (Armour et al., 2009) with slight modification of the PCR amplification cycle of. For the NSR-primed cDNA synthesis, 2 µL of 100 µmol/L first-strand NSR primer mix were mixed with 1 µL of total RNA, 1 µL diluted Spike-In Mix and 6 µL of nuclear-free water in a PCR tube. The mix was heated at 65°C for 5 min and chilled on ice before adding 10 µL of high dNTP reverse transcription master mix (3 µL of water, 4 µL of 5× buffer, 1 µL of 100 mmol/L DTT, 1 µL of 40 mmol/L dNTPs and 1 µL of SuperScriptIII enzyme (Invitrogen)). The 20 µL reverse transcription reaction was incubated at 45°C for 30 min, 70°C for 15 min and cooled to 4°C. The RNA template was removed by adding 1 µL of RNase H (Invitrogen) and incubating at 37°C for 20 min, 75°C for 15min and cooling to 4°C. The cDNA was further purified using the QIAquick PCR Purification kit. For second-strand synthesis, 25 µL of purified cDNA was added to 65 µL of Klenow master mix and 10 µL of 100 µmol/L second-strand NSR primer mix. The 100 µL reaction was incubated at 37°C for 30 min and cooled to 4°C. The DNA was purified using QIAquick spin columns and eluted with 30 µL of elution buffer. For PCR amplification, 25 µL of purified second-strand synthesis reaction was combined with 75 µL of PCR master mix (19 µL of water, 20 µL of 5× Buffer 2, 10 µL of 25 mmol/L MgCl_2_, 5 µL of 10 mmol/L dNTPs, 10 µL of 10 µmol/L forward primer, 10 µL of 10 µmol/L reverse primer, 1 µL of Expand^PLUS^ enzyme (Roche)). The samples were denatured for 2 min at 94°C and followed by 2 cycles of 94°C for 10 s, 45°C for 2 min, 72°C for 1 min; 6 cycles of 94°C for 10 s, 60°C for 30 s, 72°C for 1min; 10 cycles of 94°C for 15 s, 60°C for 30 s, 72°C for 1 min with an additional 15 s added at each cycle; and 72°C for 5 min and cooling to 4°C. Double-stranded DNA was purified by QIAquick spin columns and eluted with 30 µL of elution buffer.

### Quantitative RT-PCR assay

Expression levels of the ncRNAs were evaluated using quantitative RT-PCR (qRT-PCR) assay. The assay was performed with TransScript II Green One-Step qRT-PCR Super Mix (TransGen) using a CFX96 Real-Time PCR Detection System (Bio-Rad). 50°C for 5 min for reverse transcription reaction and denatured at 94°C for 30 s, followed by 40 cycles of 94°C for 10 s, 60°C for 15 s, 72°C for 10 s. The experiments were carried out for three times for each ncRNA. The relative quantification of ncRNA expression was determined using the 2^ΔΔ^Ct method. The fold change in expression was obtained by normalizing to an internal control gene *TBG-1*. All primers used are listed in Table S12.

### Computational analysis

These NSR-Seq libraries were sequenced on Illumina GAII. An average of 30 million reads per sample was generated, with sequence lengths of 80 nt. The ERCC spike-in RNAs (http://tools.invitrogen.com/downloads/ERCC92.fa) were “added to” the *C. elegans* genome (WS190) before aligning the sequencing reads using Bowtie. The RPKM (reads per kilobase of exon per million) was computed for each gene and synthetic spike-in RNA. Reference values of Spike-In Mix transcripts are available at (http://tools.invitrogen.com/downloads/ERCC_Controls_Analysis.txt). ncRNA annotations were obtained from Refseq and Wormbase (WS190).

## Electronic supplementary material

Below is the link to the electronic supplementary material.

Supplementary Table 1. The rRNA transcripts used to filter the NSR primer set. (xls) (XLSX 10 kb)

Supplementary Table 2. The antisense and sense NSR primers set. (xls) (XLSX 29 kb)

Supplementary Table 3. cDNA PCR primers compatible with GAII sequencing. (xls) (XLSX 8 kb)

Supplementary Table 4. Mapped reads in each sample. (PDF 88 kb)

Supplementary Table 5. Reads mapped to Spike-In transcripts in each sample (PDF 94 kb)

Supplementary Table 6. The CEP-1 activated and repressed ncRNAs under normal conditions. (xls) (XLSX 27 kb)

Supplementary Table 7. The CEP-1 repressed ncRNAs under normal conditions. (xls) (XLSX 75 kb)

Supplementary Table 8. The 19 cluster of CEP-1 repressed ncRNAs. (xls) (XLSX 17 kb)

Supplementary Table 9. The CEP-1 activated and repressed ncRNAs in response to UV irradiation. (xls) (XLSX 31 kb)

Supplementary Table 10. The CEP-1 repressed ncRNAs in response to UV irradiation. (xls) (XLSX 32 kb)

Supplementary Table 11. The 96 ncRNAs whose expression status shifted from repressed to activated in response to UV. (xls) (XLSX 12 kb)

Supplementary Table 12. All primers used in the quantitative RT-PCR (qRT-PCR) assays. (xls) (XLSX 11 kb)

Supplementary Figure 1. The expression of RNase MRP RNA (mrpr-1) and two SRP RNA in 4 samples. Supplementary Figure 2. The expression of snoRNP gene *GAR-1* validated by qRT-PCR in 4 samples. Supplementary Figure 3. The expression of 5 SL2 RNA in 4 samples. Supplementary Figure 4. The validation of expression of 2 CEP-1 repressed ncRNAs cluster under normal condition. (PDF 260 kb)

## Abbreviations

ncRNAs: non-coding RNAs
NGS: next-generation sequencing
NSR: not-so-random
TERT: telomerase reverse transcriptase
TR: telomerase RNA

## References

[CR1] Adriaenssens E, Dumont L, Lottin S, Bolle D, Lepretre A, Delobelle A, Bouali F, Dugimont T, Coll J, Curgy JJ (1998). H19 overexpression in breast adenocarcinoma stromal
cells is associated with tumor values and steroid receptor status but
independent of p53 and Ki-67 expression. Am J Pathol.

[CR2] Agostini M, Tucci P, Chen H, Knight RA, Bano D, Nicotera P, McKeon F, Melino G (2010). p73 regulates maintenance of neural stem
cell. Biochem Biophys Res Commun.

[CR3] Aird D, Ross MG, Chen WS, Danielsson M, Fennell T, Russ C, Jaffe DB, Nusbaum C, Gnirke A (2011). Analyzing and minimizing PCR amplification bias in
Illumina sequencing libraries. Genome Biol.

[CR4] Allen MA, Hillier LW, Waterston RH, Blumenthal T (2011). A global analysis of *C.
elegans* trans-splicing. Genome Res.

[CR5] Armour CD, Castle JC, Chen R, Babak T, Loerch P, Jackson S, Shah JK, Dey J, Rohl CA, Johnson JM (2009). Digital transcriptome profiling using selective
hexamer priming for cDNA synthesis. Nat Methods.

[CR6] Berkers CR, Maddocks OD, Cheung EC, Mor I, Vousden KH (2013). Metabolic regulation by p53 family
members. Cell Metab.

[CR7] Boominathan L (2010). The tumor suppressors p53, p63, and p73 are
regulators of MicroRNA processing complex. PloS one.

[CR8] *C. elegans* Sequencing Consortium (1998) Genome sequence of the nematode *C. elegans*: a platform for investigating biology. Science 282: 2012–2018 10.1126/science.282.5396.2012 9851916

[CR9] Christov CP, Trivier E, Krude T (2008). Noncoding human Y RNAs are overexpressed in tumours
and required for cell proliferation. Br J Cancer.

[CR10] Crighton D, Woiwode A, Zhang C, Mandavia N, Morton JP, Warnock LJ, Milner J, White RJ, Johnson DL (2003). p53 represses RNA polymerase III transcription by
targeting TBP and inhibiting promoter occupancy by TFIIIB. Embo J.

[CR11] Deng W, Zhu XP, Skogerbo G, Zhao Y, Fu Z, Wang YD, He HS, Cai L, Sun H, Liu CN (2006). Organization of the *Caenorhabditis elegans* small non-coding transcriptome:
Genomic features, biogenesis, and expression. Genome Res.

[CR12] Derry WB, Putzke AP, Rothman JH (2001). *Caenorhabditis elegans* p53: role in
apoptosis, meiosis, and stress resistance. Science.

[CR13] Derry WB, Bierings R, van Iersel M, Satkunendran T, Reinke V, Rothman JH (2007). Regulation of developmental rate and germ cell
proliferation in *Caenorhabditis elegans*
by the p53 gene network. Cell Death Differ.

[CR14] Dugimont T, Montpellier C, Adriaenssens E, Lottin S, Dumont L, Iotsova V, Lagrou C, Stehelin D, Coll J, Curgy JJ (1998). The H19 TATA-less promoter is efficiently repressed
by wild-type tumor suppressor gene product p53. Oncogene.

[CR15] Flores ER, Tsai KY, Crowley D, Sengupta S, Yang A, McKeon F, Jacks T (2002). p63 and p73 are required for p53-dependent apoptosis
in response to DNA damage. Nature.

[CR16] Fong YY, Bender L, Wang WC, Strome S (2002). Regulation of the different chromatin states of
autosomes and X chromosomes in the germ line of *C.
elegans*. Science.

[CR17] Green DR, Chipuk JE (2006). p53 and metabolism: inside the TIGAR. Cell.

[CR18] Guttman M, Rinn JL (2012). Modular regulatory principles of large non-coding
RNAs. Nature.

[CR19] Guttman M, Amit I, Garber M, French C, Lin MF, Feldser D, Huarte M, Zuk O, Carey BW, Cassady JP (2009). Chromatin signature reveals over a thousand highly
conserved large non-coding RNAs in mammals. Nature.

[CR20] He H, Wang J, Liu T, Liu XS, Li T, Wang Y, Qian Z, Zheng H, Zhu X, Wu T (2007). Mapping the *C.
elegans* noncoding transcriptome with a whole-genome tiling
microarray. Genome Res.

[CR21] Ho J, Benchimol S (2003). Transcriptional repression mediated by the p53
tumour suppressor. Cell Death Diff.

[CR22] Huarte M, Rinn JL (2010). Large non-coding RNAs: missing links in
cancer?. Hum Mol Genet.

[CR23] Huarte M, Guttman M, Feldser D, Garber M, Koziol MJ, Kenzelmann-Broz D, Khalil AM, Zuk O, Amit I, Rabani M (2010). A large intergenic noncoding RNA induced by p53
mediates global gene repression in the p53 response. Cell.

[CR24] Hung T, Wang YL, Lin MF, Koegel AK, Kotake Y, Grant GD, Horlings HM, Shah N, Umbricht C, Wang P (2011). Extensive and coordinated transcription of noncoding
RNAs within cell-cycle promoters. Nat Genet.

[CR25] Huyen Y, Jeffrey PD, Derry WB, Rothman JH, Pavletich NP, Stavridi ES, Halazonetis TD (2004). Structural differences in the DNA binding domains of
human p53 and its *C. elegans* ortholog
Cep-1. Structure.

[CR26] Jiang L, Schlesinger F, Davis CA, Zhang Y, Li R, Salit M, Gingeras TR, Oliver B (2011). Synthetic spike-in standards for RNA-seq
experiments. Genome Res.

[CR27] Jones SJM, Riddle DL, Pouzyrev AT, Velculescu VE, Hillier L, Eddy SR, Stricklin SL, Baillie DL, Waterston R, Marra MA (2001). Changes in gene expression associated with
developmental arrest and longevity in *Caenorhabditis
elegans*. Genome Res.

[CR28] Jones TA, Otto W, Marz M, Eddy SR, Stadler PF (2009). A survey of nematode SmY RNAs. RNA Biol.

[CR29] Jung MS, Yun J, Chae HD, Kim JM, Kim SC, Choi TS, Shin DY (2001). p53 and its homologues, p63 and p73, induce a
replicative senescence through inactivation of NF-Y transcription
factor. Oncogene.

[CR30] Kedde M, le Sage C, Duursma A, Zlotorynski E, van Leeuwen B, Nijkamp W, Beijersbergen R, Agami R (2006). Telomerase-independent regulation of ATR by human
telomerase RNA. J Biol Chem.

[CR31] Kelly WG, Schaner CE, Dernburg AF, Lee MH, Kim SK, Villeneuve AM, Reinke V (2002). X-chromosome silencing in the germline of *C. elegans*. Development.

[CR32] Kenzelmann Broz D, Spano Mello S, Bieging KT, Jiang D, Dusek RL, Brady CA, Sidow A, Attardi LD (2013). Global genomic profiling reveals an extensive
p53-regulated autophagy program contributing to key p53
responses. Genes Dev.

[CR33] Kirkpatrick KL, Mokbel K (2001). The significance of human telomerase reverse
transcriptase (hTERT) in cancer. Eur J Surg Oncol.

[CR34] Krastev DB, Slabicki M, Paszkowski-Rogacz M, Hubner NC, Junqueira M, Shevchenko A, Mann M, Neugebauer KM, Buchholz F (2011). A systematic RNAi synthetic interaction screen
reveals a link between p53 and snoRNP assembly. Nat Cell Biol.

[CR35] Labbe JC, Hekimi S, Rokeach LA (1999). The levels of the RoRNP-associated Y RNA are
dependent upon the presence of ROP-1, the *Caenorhabditis elegans* Ro60 protein. Genetics.

[CR36] Labbe JC, Burgess J, Rokeach LA, Hekimi S (2000). ROP-1, an RNA quality-control pathway component,
affects *Caenorhabditis elegans dauer*
formation. Proc Natl Acad Sci USA.

[CR37] Leonova KI, Brodsky L, Lipchick B, Pal M, Novototskaya L, Chenchik AA, Sen GC, Komarova EA, Gudkov AV (2013). p53 cooperates with DNA methylation and a suicidal
interferon response to maintain epigenetic silencing of repeats and
noncoding RNAs. Proc Natl Acad Sci USA.

[CR38] Levine AJ, Tomasini R, McKeon FD, Mak TW, Melino G (2011). The p53 family: guardians of maternal
reproduction. Nat Rev Mol Cell Biol.

[CR39] Li H, Cao Y, Berndt MC, Funder JW, Liu JP (1999). Molecular interactions between telomerase and the
tumor suppressor protein p53 in vitro. Oncogene.

[CR40] Li A, Wei G, Wang Y, Zhou Y, Zhang XE, Bi L, Chen R (2012). Identification of intermediate-size non-coding RNAs
involved in the UV-induced DNA damage response in *C.
elegans*. PloS one.

[CR41] Loven J, Orlando DA, Sigova AA, Lin CY, Rahl PB, Burge CB, Levens DL, Lee TI, Young RA (2012). Revisiting global gene expression
analysis. Cell.

[CR42] Lowe J, Shatz M, Resnick M, Menendez D (2013). Modulation of immune responses by the tumor
suppressor p53. BioDiscovery.

[CR43] Lu ZJ, Yip KY, Wang G, Shou C, Hillier LW, Khurana E, Agarwal A, Auerbach R, Rozowsky J, Cheng C (2011). Prediction and characterization of noncoding RNAs in
*C. elegans* by integrating
conservation, secondary structure, and high-throughput sequencing and array
data. Genome Res.

[CR44] Lu KH, Li W, Liu XH, Sun M, Zhang ML, Wu WQ, Xie WP, Hou YY (2013). Long non-coding RNA MEG3 inhibits NSCLC cells
proliferation and induces apoptosis by affecting p53
expression. BMC Cancer.

[CR45] MacMorris M, Kumar M, Lasda E, Larsen A, Kraemer B, Blumenthal T (2007). A novel family of *C.
elegans* snRNPs contains proteins associated with
trans-splicing. RNA-A Publ RNA Soc.

[CR46] Maiuri MC, Galluzzi L, Morselli E, Kepp O, Malik SA, Kroemer G (2010). Autophagy regulation by p53. Curr Opin Cell Biol.

[CR47] Maroney PA, Yu YT, Jankowska M, Nilsen TW (1996). Direct analysis of nematode cis- and
trans-spliceosomes: a functional role for U5 snRNA in spliced leader
addition trans-splicing and the identification of novel Sm
snRNPs. RNA-A Publ RNA Soc.

[CR48] Matouk IJ, Mezan S, Mizrahi A, Ohana P, Abu-lail R, Fellig Y, deGroot N, Galun E, Hochberg A (2010). The oncofetal H19 RNA connection: hypoxia, p53 and
cancer. BBA-Mol Cell Res.

[CR49] Melino G, Lu X, Gasco M, Crook T, Knight RA (2003). Functional regulation of p73 and p63: development
and cancer. Trends Biochem Sci.

[CR50] Menendez D, Inga A, Resnick MA (2009). The expanding universe of p53 targets. Nat Rev Cancer.

[CR51] Molchadsky A, Shats I, Goldfinger N, Pevsner-Fischer M, Olson M, Rinon A, Tzahor E, Lozano G, Zipori D, Sarig R (2008). p53 plays a role in mesenchymal differentiation
programs, in a cell fate dependent manner. PloS One.

[CR52] Mondal AM, Horikawa I, Pine SR, Fujita K, Morgan KM, Vera E, Mazur SJ, Appella E, Vojtesek B, Blasco MA (2013). p53 isoforms regulate aging- and tumor-associated
replicative senescence in T lymphocytes. J Clin Investig.

[CR53] Nam JW, Bartel DP (2012). Long noncoding RNAs in *C.
elegans*. Genome Res.

[CR54] Ou HD, Lohr F, Vogel V, Mantele W, Dotsch V (2007). Structural evolution of C-terminal domains in the
p53 family. EMBO J.

[CR55] Riley T, Sontag E, Chen P, Levine A (2008). Transcriptional control of human p53-regulated
genes. Nat Rev Mol Cell Biol.

[CR56] Rinn JL, Loewer S, Huarte M, Cabili M, Guttman M, Regev A, Lander ES, Daley GQ, Rinn JL (2011) Large intergenic non-coding RNAs in chromatin, cancer and stem cells. FASEB J 25

[CR57] Roberts A, Trapnell C, Donaghey J, Rinn JL, Pachter L (2011). Improving RNA-Seq expression estimates by correcting
for fragment bias. Genome Biol.

[CR58] Roger L, Gadea G, Roux P (2006). Control of cell migration: a tumour suppressor
function for p53?. Biol Cell Under Auspices Eur Cell Biol Organ.

[CR59] Sabapathy K, Klemm M, Jaenisch R, Wagner EF (1997). Regulation of ES cell differentiation by functional
and conformational modulation of p53. EMBO J.

[CR60] Schumacher B, Hofmann K, Boulton S, Gartner A (2001). The *C. elegans*
homolog of the p53 tumor suppressor is required for DNA damage-induced
apoptosis. Curr Biol.

[CR61] Senoo M, Pinto F, Crum CP, McKeon F (2007). p63 is essential for the proliferative potential of
stem cells in stratified epithelia. Cell.

[CR62] Stergiou L, Doukoumetzidis K, Sendoel A, Hengartner MO (2007). The nucleotide excision repair pathway is required
for UV-C-induced apoptosis in *Caenorhabditis
elegans*. Cell Death Diff.

[CR63] Stiewe T, Zimmermann S, Frilling A, Esche H, Putzer BM (2002). Transactivation-deficient Delta TA-p73 acts as an
oncogene. Cancer Res.

[CR64] Suzuki HI, Yamagata K, Sugimoto K, Iwamoto T, Kato S, Miyazono K (2009). Modulation of microRNA processing by
p53. Nature.

[CR65] Venter JC, Adams MD, Myers EW (2001). The sequence of the human genome. Science.

[CR66] Wang Y, Chen J, Wei G, He H, Zhu X, Xiao T, Yuan J, Dong B, He S, Skogerbo G (2011). The *Caenorhabditis
elegans* intermediate-size transcriptome shows high degree of
stage-specific expression. Nucleic Acids Res.

[CR67] Xiao T, Wang Y, Luo H, Liu L, Wei G, Chen X, Sun Y, Chen X, Skogerbo G, Chen R (2012). A differential sequencing-based analysis of the
*C. elegans* noncoding
transcriptome. RNA.

[CR68] Xu DW, Wang Q, Gruber A, Bjorkholm M, Chen ZG, Zaid A, Selivanova G, Peterson C, Wiman KG, Pisa P (2000). Downregulation of telomerase reverse transcriptase
mRNA expression by wild type p53 in human tumor cells. Oncogene.

[CR69] Yang A, McKeon F (2000). P63 and P73: P53 mimics, menaces and
more. Nat Rev Mol Cell Biol.

[CR70] Yang A, Zhu Z, Kettenbach A, Kapranov P, McKeon F, Gingeras TR, Struhl K (2010). Genome-wide mapping indicates that p73 and p63
co-occupy target sites and have similar dna-binding profiles in
vivo. PloS One.

[CR71] Yang F, Bi J, Xue X, Zheng L, Zhi K, Hua J, Fang G (2012). Up-regulated long non-coding RNA H19 contributes to
proliferation of gastric cancer cells. FEBS J.

[CR72] Yu X, Harris SL, Levine AJ (2006). The regulation of exosome secretion: a novel
function of the p53 protein. Cancer Res.

[CR73] Zhang L, Yu D, Hu M, Xiong S, Lang A, Ellis LM, Pollock RE (2000). Wild-type p53 suppresses angiogenesis in human
leiomyosarcoma and synovial sarcoma by transcriptional suppression of
vascular endothelial growth factor expression. Cancer Res.

[CR74] Zhou Y, Zhong Y, Wang Y, Zhang X, Batista DL, Gejman R, Ansell PJ, Zhao J, Weng C, Klibanski A (2007). Activation of p53 by MEG3 non-coding
RNA. J Biol Chem.

