# Supplementary material for: Analysis of the p53/CEP-1 regulated non-coding transcriptome in C. elegans by an NSR-seq strategy
Source: Protein Cell. 2014 May 21;5(10):770–82. doi: 10.1007/s13238-014-0071-y (PMC4180458; doi:10.1007/s13238-014-0071-y)
Supplement: Supplementary file 4 — Supplementary Table 4. Mapped reads in each sample. (PDF 88 kb) [file 13238_2014_71_MOESM4_ESM.pdf]

Supplementary Table 4: percentage of the mapped reads in samples.

Sample	Total_reads	Mapped reads	Mapped percentage
N2	29,041,373	16,873,187	58.10%
N2/UV	30,020,895	18,204,059	60.64%
cep-1	26,220,987	17,153,170	65.42%
cep-1/UV	28,728,812	18,203,104	63.36%
cep-1_rep	18,640,764	12,567,062	67.42%

Supplementary Table 5: percentage of the mapped Spike-In transcripts in samples.

Sample	Total_reads	Mapped to Spike-In reads	Spike-In Percentage
N2	29,041,373	64,719	0.22%
N2/UV	30,020,895	74,766	0.25%
cep-1	26,220,987	71,262	0.27%
cep-1/UV	28,728,812	84,168	0.29%
cep-1_rep	18,640,764	89,385	0.48%
