# Supplementary material for: Analysis of the p53/CEP-1 regulated non-coding transcriptome in C. elegans by an NSR-seq strategy
Source: Protein Cell. 2014 May 21;5(10):770–82. doi: 10.1007/s13238-014-0071-y (PMC4180458; doi:10.1007/s13238-014-0071-y)
Supplement: Supplementary file 5 — Supplementary Table 5. Reads mapped to Spike-In transcripts in each sample (PDF 94 kb) [file 13238_2014_71_MOESM5_ESM.pdf]

Supplementary Table 5: percentage of the mapped Spike-In transcripts in samples.

Sample	Total_reads	Mapped to Spike-In reads	Spike-In Percentage
N2	29,041,373	64,719	0.22%
N2/UV	30,020,895	74,766	0.25%
cep-1	26,220,987	71,262	0.27%
cep-1/UV	28,728,812	84,168	0.29%
cep-1_rep	18,640,764	89,385	0.48%
