# Supplementary material for: Analysis of the p53/CEP-1 regulated non-coding transcriptome in C. elegans by an NSR-seq strategy
Source: Protein Cell. 2014 May 21;5(10):770–82. doi: 10.1007/s13238-014-0071-y (PMC4180458; doi:10.1007/s13238-014-0071-y)

Supplementary Figure 1. The expression of RNase MRP RNA (*MRPR-1*) and two SRP RNA in 4 samples.

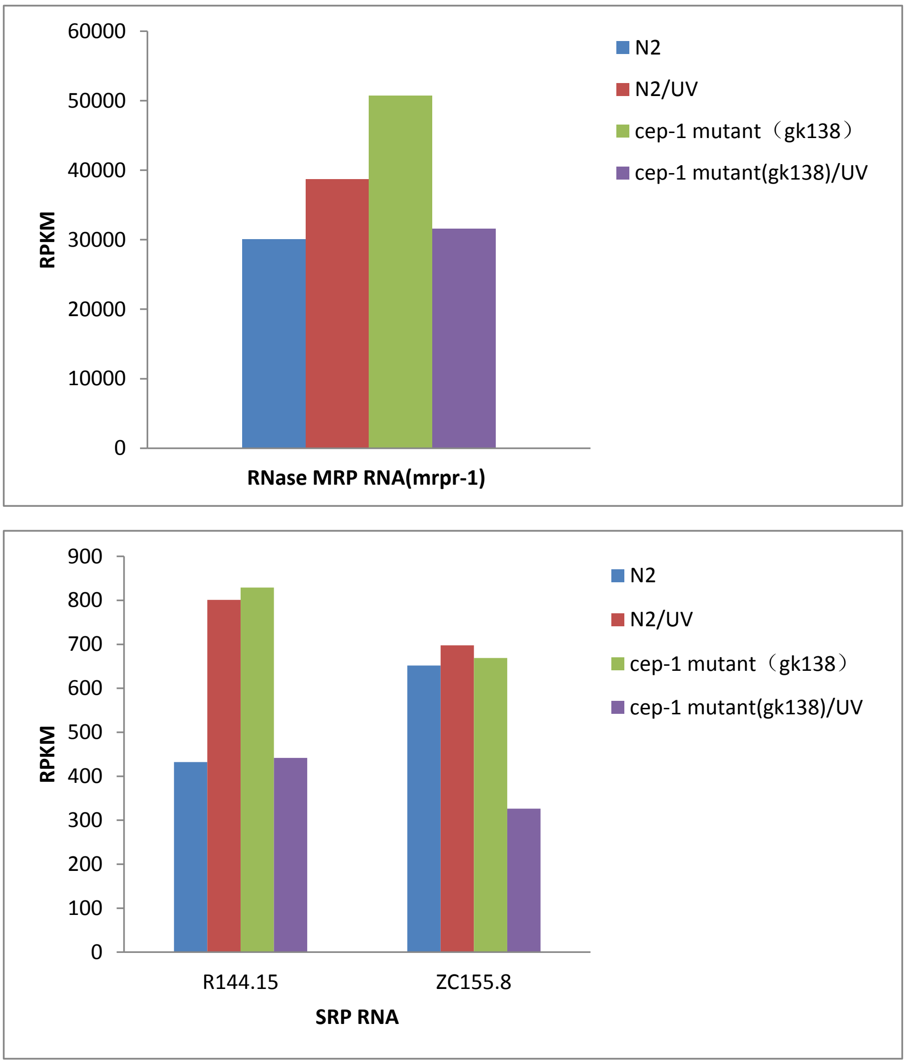

Supplementary Figure 2. The expression of snoRNP gene *GAR-1* validated by qRT-PCR in 4 samples.

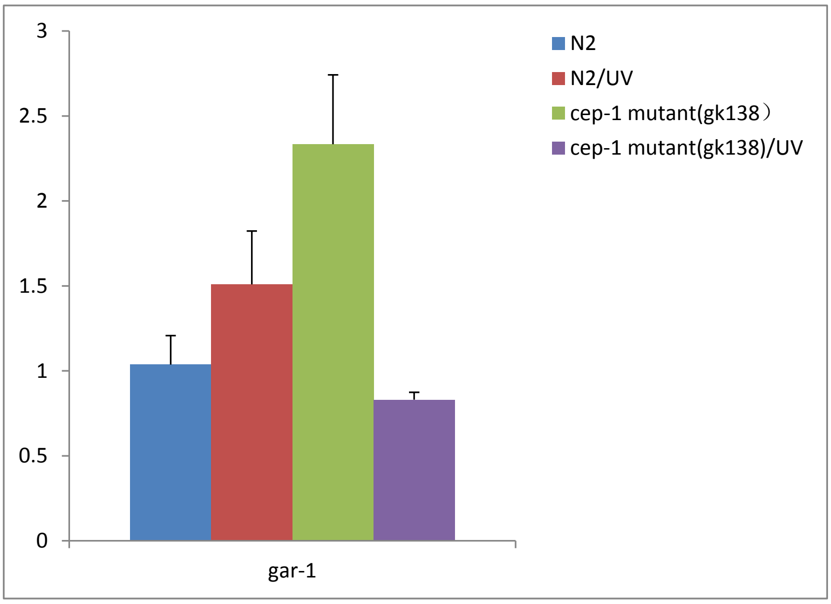

Supplementary Figure 3. The expression of 5 SL2 RNA in 4 samples.

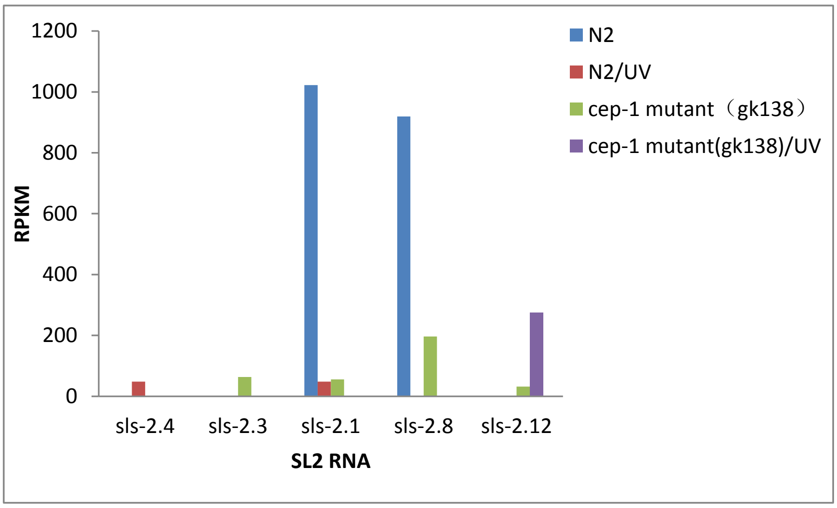

Supplementary Figure 4: The validation of expression of 2 CEP-1 repressed ncRNAs cluster under normal condition.

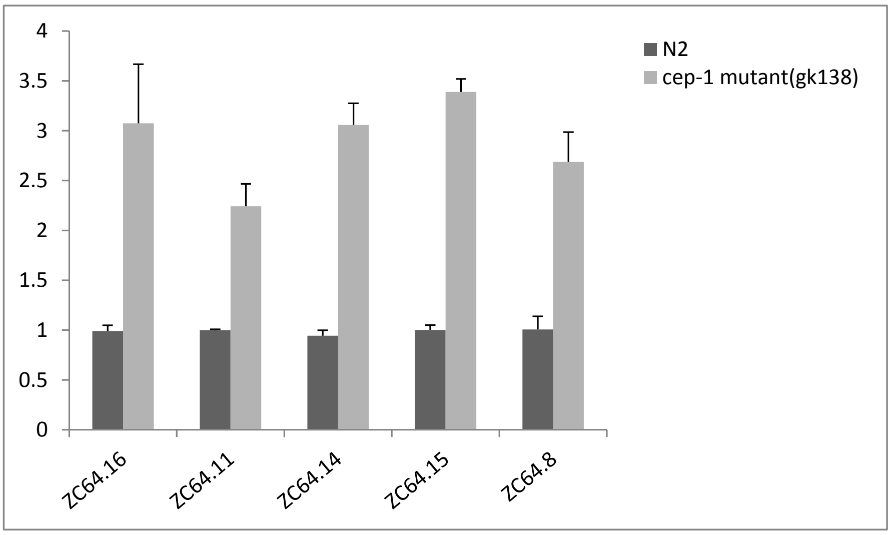

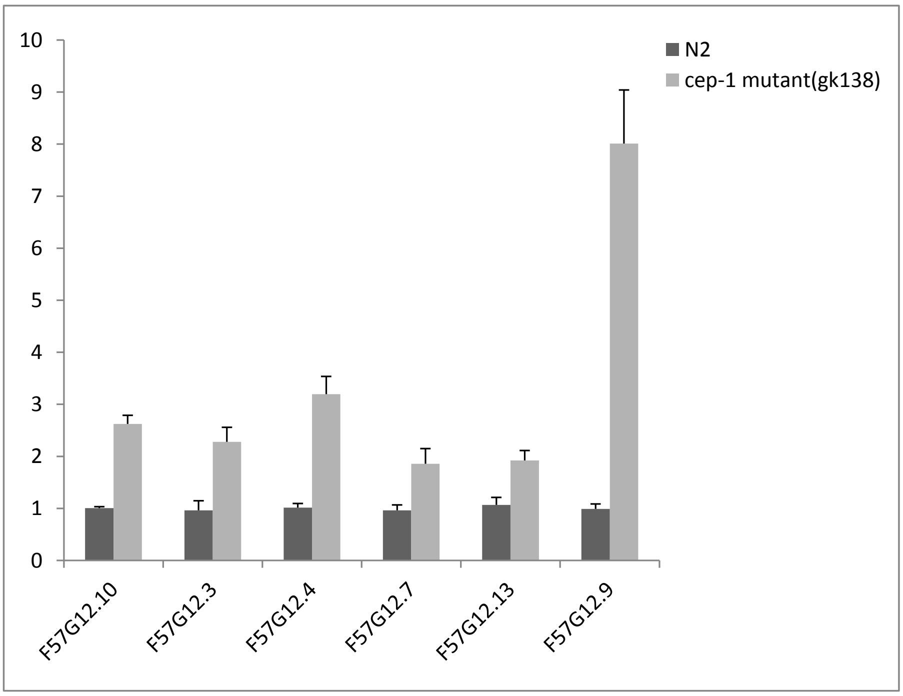

Supplement: Supplementary file 13 — Supplementary Figure 1. The expression of RNase MRP RNA (mrpr-1) and two SRP RNA in 4 samples. Supplementary Figure 2. The expression of snoRNP gene GAR-1 validated by qRT-PCR in 4 samples. Supplementary Figure 3. The expression of 5 SL2 RNA in 4 samples. Supplementary Figure 4. The validation of expression of 2 CEP-1 repressed ncRNAs cluster under normal condition. (PDF 260 kb) [file 13238_2014_71_MOESM13_ESM.pdf]
